# Ubiquitin-specific protease 38 promotes inflammatory atrial fibrillation induced by pressure overload

**DOI:** 10.1093/europace/euad366

**Published:** 2024-01-29

**Authors:** Zheng Xiao, Yucheng Pan, Bin Kong, Hong Meng, Wei Shuai, He Huang

**Affiliations:** Department of Cardiology, Renmin Hospital of Wuhan University, 238 Jiefang Road, Wuhan 430060, Hubei, China; Cardiovascular Research Institute of Wuhan University, Wuhan 430060, Hubei, China; Hubei Key Laboratory of Cardiology, Wuhan 430060, Hubei, China; Department of Cardiology, Renmin Hospital of Wuhan University, 238 Jiefang Road, Wuhan 430060, Hubei, China; Cardiovascular Research Institute of Wuhan University, Wuhan 430060, Hubei, China; Hubei Key Laboratory of Cardiology, Wuhan 430060, Hubei, China; Department of Cardiology, Renmin Hospital of Wuhan University, 238 Jiefang Road, Wuhan 430060, Hubei, China; Cardiovascular Research Institute of Wuhan University, Wuhan 430060, Hubei, China; Hubei Key Laboratory of Cardiology, Wuhan 430060, Hubei, China; Department of Cardiology, Renmin Hospital of Wuhan University, 238 Jiefang Road, Wuhan 430060, Hubei, China; Cardiovascular Research Institute of Wuhan University, Wuhan 430060, Hubei, China; Hubei Key Laboratory of Cardiology, Wuhan 430060, Hubei, China; Department of Cardiology, Renmin Hospital of Wuhan University, 238 Jiefang Road, Wuhan 430060, Hubei, China; Cardiovascular Research Institute of Wuhan University, Wuhan 430060, Hubei, China; Hubei Key Laboratory of Cardiology, Wuhan 430060, Hubei, China; Department of Cardiology, Renmin Hospital of Wuhan University, 238 Jiefang Road, Wuhan 430060, Hubei, China; Cardiovascular Research Institute of Wuhan University, Wuhan 430060, Hubei, China; Hubei Key Laboratory of Cardiology, Wuhan 430060, Hubei, China

**Keywords:** Ubiquitin-specific protease 38, Cardiac remodelling, Atrial fibrillation, Inflammation

## Abstract

**Aims:**

Atrial structural and electrical remodelling is a major reason for the initiation and perpetuation of atrial fibrillation (AF). Ubiquitin-specific protease 38 (USP38) is a deubiquitinating enzyme, but its function in the heart remains unknown. The aim of this study was to investigate the effect of USP38 in pressure overload-induced AF.

**Methods and results:**

Cardiac-specific knockout USP38 and cardiac-specific transgenic USP38 mice and their corresponding control mice were used in this study. After 4 weeks with or without aortic banding (AB) surgery, atrial echocardiography, atrial histology, electrophysiological study, and molecular analysis were assessed. Ubiquitin-specific protease 38 knockout mice showed a remarkable improvement in vulnerability to AF, atrial weight and diameter, atrial fibrosis, and calcium-handling protein expression after AB surgery. Conversely, USP38 overexpression further increased susceptibility to AF by exacerbating atrial structural and electrical remodelling. Mechanistically, USP38 interacted with and deubiquitinated nuclear factor-kappa B (NF-κB), and USP38 overexpression increased the level of p-NF-κB *in vivo* and *in vitro*, accompanied by the upregulation of NOD-like receptor protein 3 (NLRP3) and inflammatory cytokines, suggesting that USP38 contributes to adverse effects by driving NF-κB/NLRP3-mediated inflammatory responses.

**Conclusion:**

Overall, our study indicates that USP38 promotes pressure overload-induced AF through targeting NF-κB/NLRP3-mediated inflammatory responses.

## Graphical Abstract

**Graphical Abstract:**
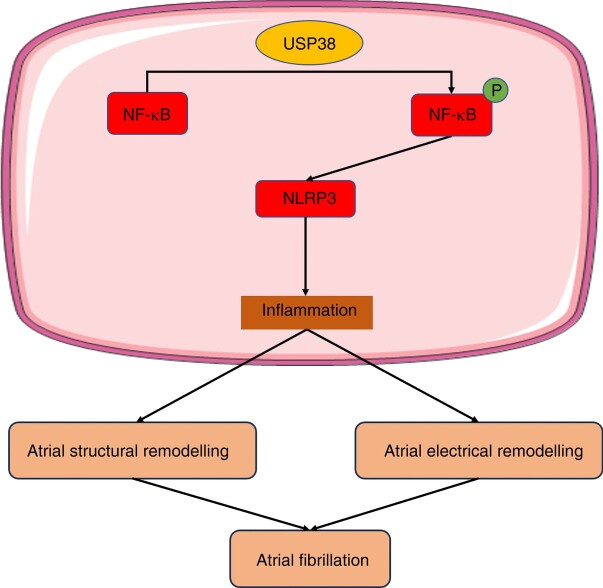

**What’s new?**

- Ubiquitin-specific protease 38 (USP38) was upregulated in the left atrium after aortic banding surgery.
- Cardiac-specific knockout of USP38 attenuated the atrial dilation and fibrosis and reduced the vulnerability of atrial fibrillation (AF) induced by pressure overload.
- Cardiac-specific overexpression of USP38 aggravated the atrial structural and electrical remodelling induced by pressure overload.
- Ubiquitin-specific protease 38 promoted atrial inflammation by interacting with and deubiquitinating nuclear factor-kappa B.
- Targeting USP38 may be an effective treatment for AF induced by pressure overload.

## Introduction

Atrial fibrillation (AF) is the most common cardiac arrhythmia in clinics, affecting approximately 1.6% population in China, and leads to stroke and severe public health burden.^1^ Although there has been some progress in drug treatment for AF, its further development has been hampered by extra-cardiac toxicities and pro-arrhythmic effects.

Under chronic pressure overload stimulation, atrial structural and electrical changes are known as remodelling. In recent years, the pathogenesis of AF mainly focuses on cardiac remodelling, including atrial fibrosis, abnormal expression of ion channel proteins, and inflammation.^2^ There is growing evidence that inflammation is a key marker of AF.^3–5^ The levels of pro-inflammation cytokines in patients with AF are increased and greatly affect the prognosis of patients with AF.^6^ In addition, a recent study has shown that the risk of stroke and thromboembolism in patients with AF are also associated with pro-inflammatory mediator.^7^ Nuclear factor-kappa B (NF-κB) is widely recognized as a key component in inflammation.^8^ Activation of NF-κB promotes the expression of a series of pro-inflammatory cytokines, chemokines, and adhesion molecules.^8^ Inhibition of NF-κB-mediated inflammation can exert cardioprotective effects in various heart diseases.^9,10^ Furthermore, our previous study showed that NF-κB-mediated inflammatory signalling promotes the progression of obesity-related AF.^11^ In pressure overload-induced cardiac remodelling mice, NF-κB activation exacerbates atrial inflammatory responses and increases vulnerability to AF.^12^ Recently, NOD-like receptor protein 3 (NLRP3) has gained significant attention due to its role in inflammation, with NLRP3-mediated inflammation playing a crucial role in the progression of AF.^13^ Nuclear factor-kappa B, one of the upstream signalling of NLRP3, participates in the regulation of inflammatory responses.^14^ Therefore, inhibiting NF-κB/NLRP3 signalling may prevent pressure overload-induced AF.

Ubiquitin-specific proteases (USPs) belong to the deubiquitinating enzyme family and play an important role in deubiquitinating modification. The function of USPs is to maintain a balanced level of protein under physical condition by removing ubiquitin from the substrate protein, thereby preventing the degradation of the substrate protein through the proteasome pathway.^15^ Ubiquitin-specific proteases mediate a series of physiological and pathological processes, including inflammation, autophagy, apoptosis, and ferroptosis.^16–18^ For instance, Colleran *et al*.^19^ found that USP7 can deubiquitinate NF-κB in the nucleus, thus increasing NF-κB transcriptional activity and ultimately aggravating the inflammatory response. Moreover, USP5 knockdown improves endothelial inflammation by suppressing the activation of NF-κB signalling.^20^ Recently, USP15 has been reported to be involved in regulating activation of NF-κB. Overexpression of USP15 aggravates tumour necrosis factor-α (TNF-α)- and interleukin (IL)-1β-induced NF-κB activation.^21^ Ubiquitin-specific protease 38 is a member of USPs and is involved in the process of cell proliferation, cell migration, virus infection, and inflammatory response.^22–26^ However, the role of USP38 in AF remains unknown.

In the present study, we used both loss-of-function and gain-of-function approaches to explore the molecular mechanism of USP38 in pressure overload-induced AF. Our findings revealed that USP38 deletion reduced vulnerability to AF induced by pressure overload, while overexpression of USP38 had the opposite effect. Ubiquitin-specific protease 38 interacted with NF-κB to promote the deubiquitination of NF-κB, exacerbating inflammatory response. Therefore, USP38 may be a potential target for pressure overload-induced AF.

## Methods

### Animals

The animal study complied with the National Institutes of Health Guideline on the Care and Use of Laboratory, with approval by the Animal Ethics Committee of Renmin Hospital of Wuhan University (WDRM20221207B). The construction of a genetic mouse was performed at Cyagen Biotechnology. Briefly, the single guide RNA synthesized *in vitro* was transferred into mouse zygotes to produce corresponding progeny, which were then crossed with cardiac-specific Cre tool mice for several generations to obtain homozygous cardiac-specific knockout USP38 (USP38^cko^) mice. The sequence of primers for genotypic identification of USPS38^cko^ mice is shown as follows: F: 5′-ATGATCGGAGGTTTCCTTGTGTTG-3′ and R: 5′-TCTGATGTCTGAGTATCAACGAAGA-3′. Similarly, the corresponding sgRNA was transferred into mouse zygotes to produce conditional knockin progeny, which were then hybridized with cardiac-specific Cre tool mice to maintain the overexpression of USP38 in the heart. The sequence of primers for genotypic identification of cardiac-specific transgenic USP38 (USP38-TG) mice is listed as follows: F: 5′-ATCTGCTTCCTGTTCGTTCCGAC-3′ and R: 5′-CTTTATTAGCCAGAAGTCAGATGC-3′.

### Construction of pressure overload model

According to the previously described method, we established a pressure overload-induced atrial remodelling model.^27^ Briefly, after anaesthetized with pentobarbital (40 mg/kg), male mice, 8–10 weeks age, were lapped with a 27G needle to ligate the thoracic aorta. The sham surgery was performed according to the same procedure except for aortic co-arctation. The mice were used and sacrificed 4 weeks after surgery.

### Echocardiography

Using the Vinno system with a 23 MHz liner array transducer, the procedure of transthoracic echocardiography was performed on all mice. Briefly, mice were anaesthetized using 2% isoflurane and kept warm using a heating pad. The left atrial size was measured via the parasternal long-axis view, while the left ventricular ejection fraction (LVEF) and left ventricular fractional shortening (LVFS) were evaluated via the parasternal short-axis view.

### Nuclear factor-kappa B inhibitor (pyrrolidine dithiocarbamate) administration

In vivo experiment, pyrrolidine dithiocarbamate (PDTC, Sigma, USA) was intra-peritoneally injected into USP38-TG mice at a previously reported dose (120 mg/kg/day).^28^ In cellular level, the HL-1 cells were treated with PDTC (100 µM) for 30 min.^29^

### Induction of atrial fibrillation

The mice were anaesthetized via intra-peritoneally injected 1% pentobarbital sodium. Standard surface electrocardiogram was recorded on the PowerLab System (ADInstruments). The tracheal tube was inserted into the tracheal through the glottis, and the chest fluctuation of the mice was observed to be consistent with the ventilator frequency, which proved that the tracheal intubation was successful, then, opening the cheat along the subcostal margin to fully expose the heart. The platinum stimulation electrode was placed on the appendages of the right atrium. A self-made single-phase action potential electrode was used to record the left atrium action potential. The effective refractory period (ERP) of left atria was measured with eight consecutive S1 stimuli of 100 ms pacing length and one S2 stimulus of decreasing pacing length until there was no pre-systolic wave after S2. The AF inducibility was evaluated by applying three chains of 2-s-burst pacing (100 Hz, 5 V) using stimulating electrodes. The duration of subsequent AFs after each burst pacing was recorded.

### Histological assay

The left atrial tissue was fixed in 4% paraformaldehyde and embedded in paraffin. Section of atrial tissue were cut into 5 µm and dyed with Masson’s staining. Image was acquired by light microscopy, and the level of atrial fibrosis was evaluated by Image J.

### Serum analysis

Blood samples were collected from the inferior vena cava and centrifuged for serum. The serum expression levels of the pro-inflammatory cytokines of TNF-α (GEM0004, Servicebio, Wuhan, China), IL-1β (88–7013, Invitrogen, USA), and IL-6 (GEM0001, Servicebio, Wuhan, China) were determined using Enzyme-linked immunosorbent assay Kit.

### Cell culture and transfection

Mouse atrial muscle cells, HL-1, were cultured with Claycomb Medium containing 10% FBS (10099, Gibco, Australia), 100 µM epinephrine, and 4 mM l-glutamine under a temperature of 37 °C and 5% CO_2_. After transfected with adenovirus [adenovirus-expressing short hairpin RNA (AdshRNA), adenovirus-expressing short hairpin RNA-targeting USP38 (AdshUSP38), adenovirus-expressing green fluorescent protein (AdGFP), and adenovirus-expressing USP38 (AdUSP38)], the knockdown or overexpression efficiency was evaluated by western blot. In order to mimic hypertrophic cardiomyocytes *in vitro*, cells were treated with Ang II (1 µM) for 48 h.

### Immunofluorescence

Immunofluorescence was performed according to standard procedures. HL-1 cells were fixed in 4% paraformaldehyde for 15 min and then permeabilized with 0.5% Triton X-100 (GC204003, Servicebio, Wuhan, China) for 5 min. For co-localization detection, cells were stained with the anti-USP38 antibody and corresponding secondary antibody. Then, anti-NF-κB antibody and corresponding secondary antibody were added to cells. Finally, the nucleus was stained by DAPI (G1012, Servicebio, Wuhan, China). The images were acquired via a confocal scanning microscope (NIKON Eclipse TI, Tokyo, Japan).

### Quantitative reverse transcription polymerase chain reaction

As mentioned above, the total RNA of left atrial tissue was extracted using TRIpure Total RNA extraction Reagent (EP013, ELK Biotechnology, Wuhan, China). Complementary DNA was synthesized with the help of Reverse Transcription Kit (G3329, Servicebio, Wuhan, China). SYBR Green qPCR Master Mix (G3326, Servicebio, Wuhan, China) was used to detect the level of gene expression. GAPDH acts as the internal reference. The primer sequence was presented in Supplementary material online, *Table S1*.

### Western blot

The mouse atrial tissue or HL-1 cells were lysed in RIPA buffer (G2002, Servicebio, Wuhan, China) supplemented with protease (G2008, Servicebio, Wuhan, China) and phosphatase inhibitors (G2007, Servicebio, Wuhan, China). The protein concentration was detected using the BCA Kit (G2026, Servicebio, Wuhan, China). An equal amount of protein was separated with SDS-PAGE and then transferred into PVDF membranes (Millipore, USA). After being blocked with skim milk, the membranes were incubated with corresponding primary antibody (see Supplementary material online, *Table S2*) overnight and then incubated with secondary antibody for 1 h. Finally, the protein signals were visualized using chemiluminescence (BL520A, Biosharp, Hefei, China).

### Co-immunoprecipitation

HL-1 cells were lysed using lysis buffer supplemented with PMSF and Cocktail. The cell lysates were then incubated overnight with anti-USP38, anti-NF-κB, or anti-IgG antibodies at 4 °C. Subsequently, the magnetic beads (L-1004, Biolinkedin, Shanghai, China) were placed in the cell lysates and washed with loading buffer. Finally, the samples were analysed using western blot.

### Ubiquitination assay

After adenovirus transfection, Ang II stimulation, and MG132 administration, the cell lysates from HL-1 cells were collected. Anti-NF-κB antibodies were added to cell lysates and incubated overnight at 4 °C. Then, the magnetic beads were added to cell lysates and incubated for 2 h. Finally, western blot was used to detect the ubiquitination level of NF-κB.

### Statistical analysis

For data with a Gaussian distribution, two sets of data analysis used unpaired Student’ *t*-test. Multiple group comparisons were performed by a one-way analysis of variance followed by Tukey’s multiple comparisons test. For data with a non-Gaussian distribution, we performed a non-parametric statistical analysis using the Mann–Whitney test for two sets or the Kruskal–Wallis test for multiple comparisons. The induction rate of AF was analysed by Fisher’s exact test. Normally distributed data are presented as mean ± standard deviation, and non-normally distributed data are presented as median ± interquartile range. A *P*-value of <0.05 was considered statistically significant. Statistical analysis was performed by the GraphPad Prism 9.0.0 software.

## Results

### The expression of ubiquitin-specific protease 38 is upregulated under hypertrophic stimulation *in vivo* and *in vitro*

**Figure 1:**
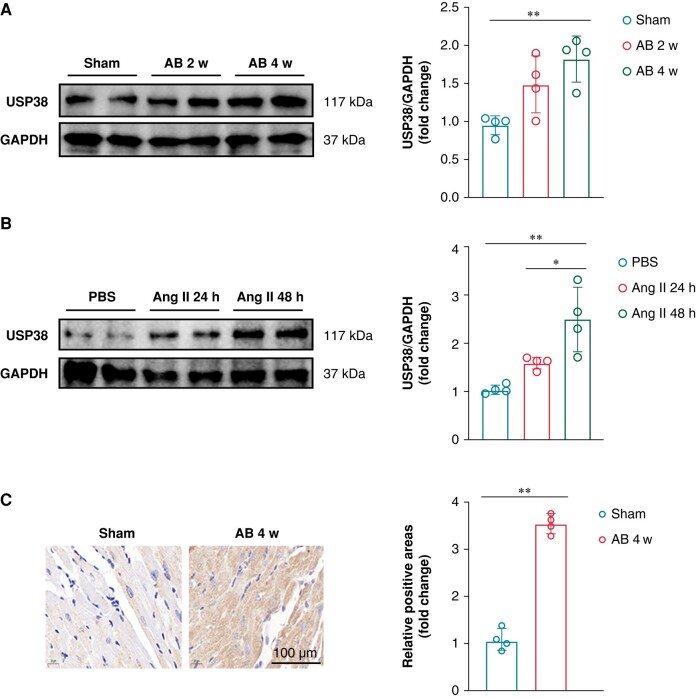
USP38 expression is upregulated in pressure overload-induced atrial tissues. (*A*) Representative western blot images and statistical analysis of the USP38 protein level in left atrial tissues from wild-type mice after AB surgery at 2 and 4 weeks (*n* = 3). (*B*) Representative western blot images and statistical analysis of the USP38 protein level in HL-1 cells 48 h after Ang II or PBS stimulation (*n* = 3). (*C*) Representative images of immunohistochemical staining and statistical analysis of the USP38 level in left atrial tissues from wild-type mice 0 and 4 weeks after AB surgery (*n* = 4). Data were calculated by one-way analysis of variance (Tukey’s multiple comparisons test) or Student’s *t*-test (unpaired, two-tailed, two groups). *P < 0.05; **P < 0.01.

To determine whether the expression of USP38 in atrial tissue was affected by hypertrophic stimulation, aortic banding (AB) surgery was performed on wild-type mice. The results demonstrated a time-dependent increase in USP38 protein levels (*Figure 1A*). Similar results were observed in HL-1 cells stimulated by Ang II (*Figure 1B*). Furthermore, immunohistochemistry analysis revealed that pressure overload increased the expression of USP38 in mouse atrial tissue (*Figure 1C*). The elevation of USP38 indicated a potential association between USP38 and atrial remodelling.

### Ubiquitin-specific protease 38 increases vulnerability to atrial fibrillation

**Figure 2:**
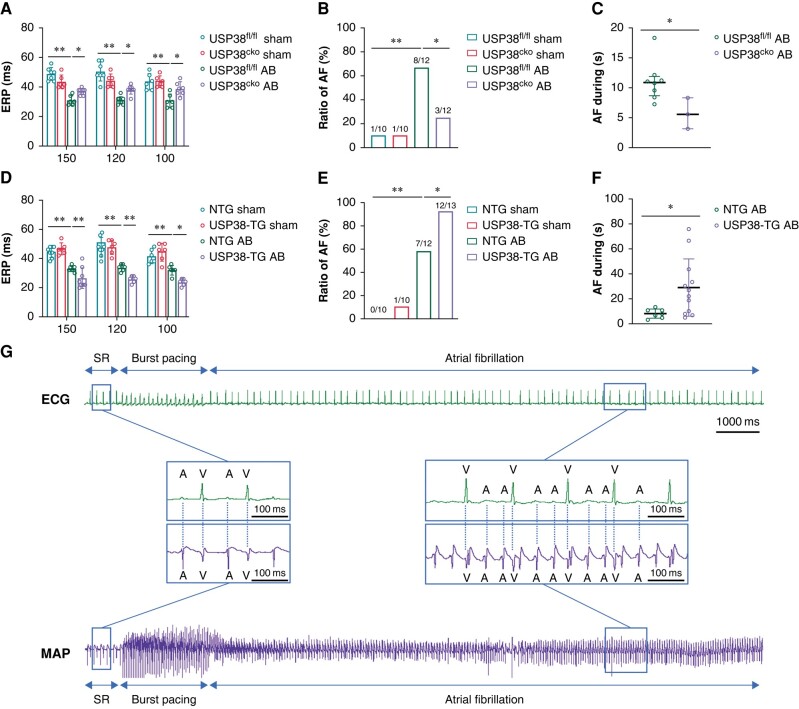
USP38 alters electrophysiological properties of the atrial in pressure overload-induced mice. (*A*) ERP of the left atrium of USP38^fl/fl^ and USP38^cko^ mice at 4 weeks after sham or AB surgery evaluated (*n* = 6–8). (*B*) Inducibility of AF of USP38^fl/fl^ and USP38^cko^ mice at 4 weeks after sham or AB surgery (*n* = 10–12). (*C*) Duration of AF of USP38^fl/fl^ and USP38^cko^ mice at 4 weeks after AB surgery (*n* = 10–12). (*D*) ERP of left atrium of NTG and USP38-TG mice at 4 weeks after sham or AB surgery evaluated (*n* = 6–8). (*E*) Inducibility of AF of NTG and USP38-TG mice at 4 weeks after sham or AB surgery (*n* = 10–13). (*F*) Duration of AF of NTG and USP38-TG mice at 4 weeks after AB surgery (*n* = 10–13). (*G*) Representative electrocardiogram (ECG) and monophasic action potential (MAP) of AF induction after burst pacing in pressure overload-induced mice. Data in *A* and *D* were calculated by one-way analysis of variance (Tukey’s multiple comparisons test). Data in *B* and *E* were calculated by Fisher’s exact test. Data in *C* and *F* was calculated by Student’s *t*-test (unpaired, two-tailed, two groups). *P < 0.05; **P < 0.01.

To investigate the functional contribution of USP38 in pressure overload-induced atrial remodelling, we constructed USP38^cko^ mice and USP38-TG mice (see Supplementary material online, *Figure S1A* and *B*). In the present study, we found that AB operation caused ERP shortening was improved in USP38^cko^ mice (*Figure 2A*). Meanwhile, pressure overload led to increased susceptibility to AF, whereas USP38 deletion was associated with a dramatic reduction in vulnerability and duration of AF induced by pressure overload (*Figure 2B* and *C*). Conversely, we found that USP38-TG mice had shorter ERP and increased vulnerability to AF with prolonged AF duration under pressure overload stimulation compared with NTG mice (*Figure 2D–F*).

### Ubiquitin-specific protease 38 deletion alleviates pressure overload-induced atrial structural remodelling

**Figure 3:**
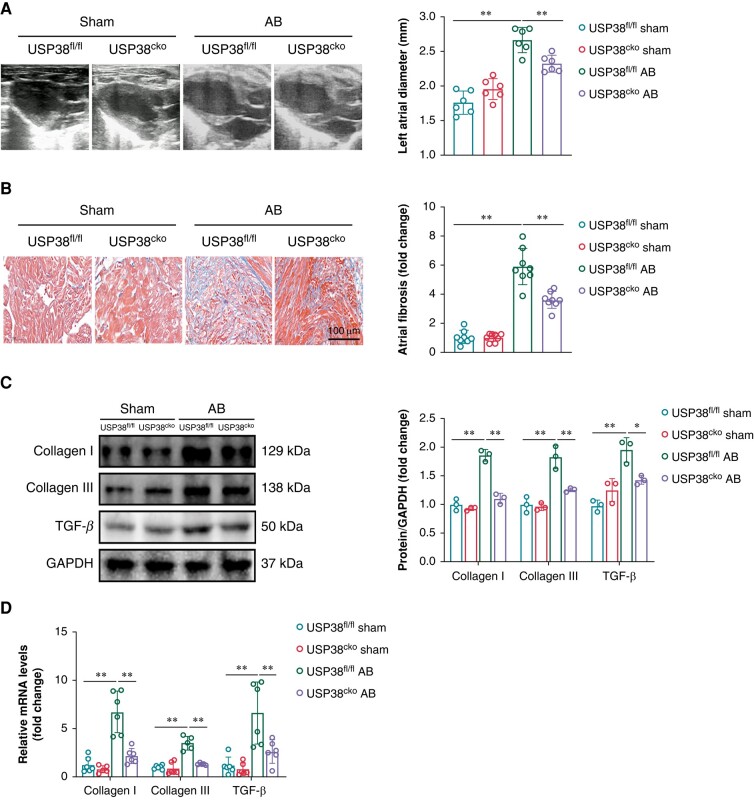
USP38 deletion improves pressure overload-induced atrial structural remodelling. (*A*) Representative echocardiographic gross images and statistical analysis of the left atrium of USP38^fl/fl^ and USP38^cko^ mice at 4 weeks after sham or AB surgery (*n* = 6–7). (*B*) Representative Masson staining images and statistical analysis of atrial tissues of the left atrium of USP38^fl/fl^ and USP38^cko^ mice at 4 weeks after sham or AB surgery (*n* = 8). (*C*) Representative western blot images and statistical analysis of the Collagen I, Collagen III, and TGF-β protein level in left atrial tissues of USP38^fl/fl^ and USP38^cko^ mice at 4 weeks after sham or AB surgery (*n* = 3). (*D*) The statistical analysis of mRNA expression of Collagen I, Collagen III, and TGF-β in left atrial tissues of USP38^fl/fl^ and USP38^cko^ mice at 4 weeks after sham or AB surgery (*n* = 5–6). Data were calculated by one-way analysis of variance (Tukey’s multiple comparisons test). *P < 0.05, **P < 0.01.

Four weeks after AB operation, the ratio of left atrial weight/tibia length was significantly increased in both groups, whereas USP38 knockdown reduced the ratio (see Supplementary material online, *Figure S2A*). Meanwhile, the enlargement of left atrial size after AB surgery was observed by echocardiography, while compared with USP38^fl/fl^ mice, a marked improvement was represented in USP38^cko^ mice, as demonstrated by decreased left atrial diameter (LAD) (*Figure 3A*). Ventricular function exhibited a similar trend, with increased LVEF and LVFS in USP38^cko^ AB mice compared with USP38^fl/fl^ AB mice (see Supplementary material online, *Figure S3A*–*C*). Consistent with the above findings, myocardial fibrosis was evident in left atrial tissues after AB surgery, whereas the degree of fibrosis was mitigated in USP38 knockout mice (*Figure 3B*). This reduction was reflected at both the transcription and protein levels, as demonstrated by mRNA and protein levels of Collagen I, Collagen III, and Transforming growth factor β (TGF-β) expressions (*Figure 3C* and *D*).

### Ubiquitin-specific protease 38 overexpression aggravates pressure overload-induced atrial structural remodelling

**Figure 4:**
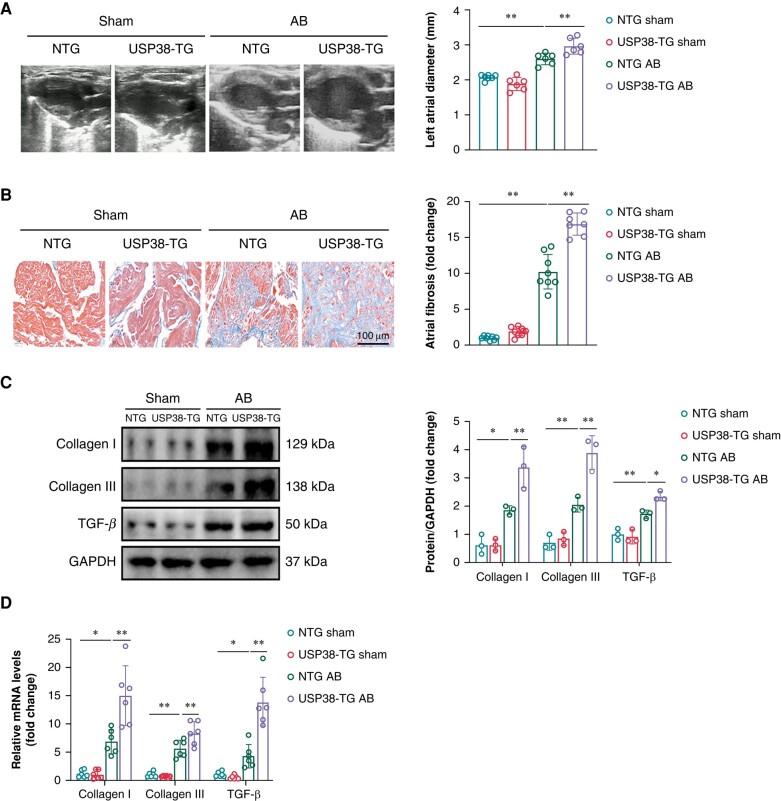
USP38 overexpression aggravates pressure overload-induced atrial structural remodelling. (*A*) Representative echocardiographic images and statistical analysis of the left atrium of NTG and USP38-TG mice at 4 weeks after sham or AB surgery (*n* = 6–7). (*B*) Representative Masson staining images and statistical analysis of atrial tissues of NTG and USP38-TG mice at 4 weeks after sham or AB surgery (*n* = 8). (*C*) Representative western blot images and statistical analysis of the Collagen I, Collagen III, and TGF-β protein level in left atrial tissues of NTG and USP38-TG mice at 4 weeks after sham or AB surgery (*n* = 3). (*D*) The statistical analysis of mRNA expression of Collagen I, Collagen III, and TGF-β in left atrial tissues of NTG and USP38-TG mice at 4 weeks after sham or AB surgery (*n* = 5–6). Data were calculated by one-way analysis of variance (Tukey’s multiple comparisons test). *P < 0.05; **P < 0.01.

Next, we observed that left atrial weight/tibia length was heavily increased in USP38-TG group compared with NTG group after AB surgery (see Supplementary material online, *Figure S2B*). Additionally, the enlargement of LAD was observed in USP38-TG group compared with NTG group under AB stimulation (*Figure 4A*). Conversely, LVEF and LVFS showed further reduction in USP38-TG AB group compared with NTG AB group (see Supplementary material online, *Figure S3D*–*F*). Furthermore, the level of atrial fibrosis and fibrosis marker gene transcription were heightened in mice with USP38 overexpression after AB surgery (*Figure 4B–D*).

### Ubiquitin-specific protease 38 deletion improves pressure overload-induced atrial electrical remodelling

**Figure 5:**
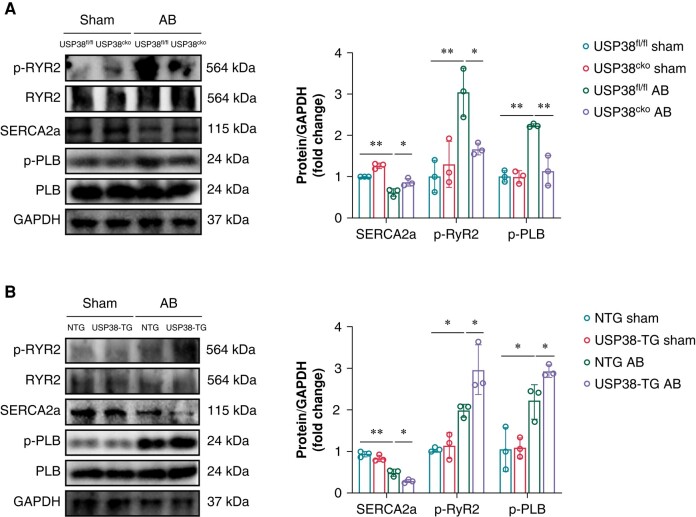
USP38 alters calcium-handling protein levels in atrial tissues. (*A*) Representative western blot images and statistical analysis of the SERCA2a, p-RyR2, and p-PLB protein level in left atrial tissues of USP38^fl/fl^ and USP38^cko^ mice at 4 weeks after sham or AB surgery (*n* = 3). (*B*) Representative western blot images and statistical analysis of the SERCA2a, p-RyR2, and p-PLB protein level in left atrial tissues of NTG and USP38-TG mice at 4 weeks after sham or AB surgery (*n* = 3). Data were calculated by one-way analysis of variance (Tukey’s multiple comparisons test). *P < 0.05; **P < 0.01.

Electrical remodelling is one of the key manifestations of cardiac remodelling. Previous studies have demonstrated that heart failure animal models exhibit abnormal function and expression in intra-cellular calcium transients and diastolic sarcoplasmic reticulum (SR) Ca^2+^ release, which is closely related to the alterations of calcium-handling proteins. Therefore, we evaluated the expression of ryanodine receptor 2 (RyR2), SR Ca^2+^-ATPase 2a (SERCA2a), and phospholamban (PLB). Similar to a previous study, the expression of p-RyR2 at Ser2808 and p-PLB at Thr17 was upregulated under pressure overload stimulation, and the adverse effect was markedly improved in USP38 knockout mice (*Figure 5A*). Furthermore, we found that pressure overload led to reduced expression of SERCA2a in atrial tissue, while USP38 deficiency mice showed an increase in the protein level of SERCA2a compared with USP38^fl/fl^ mice (*Figure 5A*). Taken together, these results suggest that USP38 deficiency improved pressure overload-induced atrial structural and electric remodelling.

### Ubiquitin-specific protease 38 overexpression accelerates pressure overload-induced atrial electrical remodelling

The USP38-TG group showed a more evident atrial electrical remodelling after AB surgery compared with the NTG group. Specifically, after AB surgery, the levels of calcium-handling proteins, increased p-RYR2 and p-PLB and decreased SERCA2a, were observed in the USP38-TG group compared with the NTG group (*Figure 5B*).

### Ubiquitin-specific protease 38 promotes inflammatory response and activates NOD-like receptor protein 3 inflammasome induced by pressure overload

**Figure 6:**
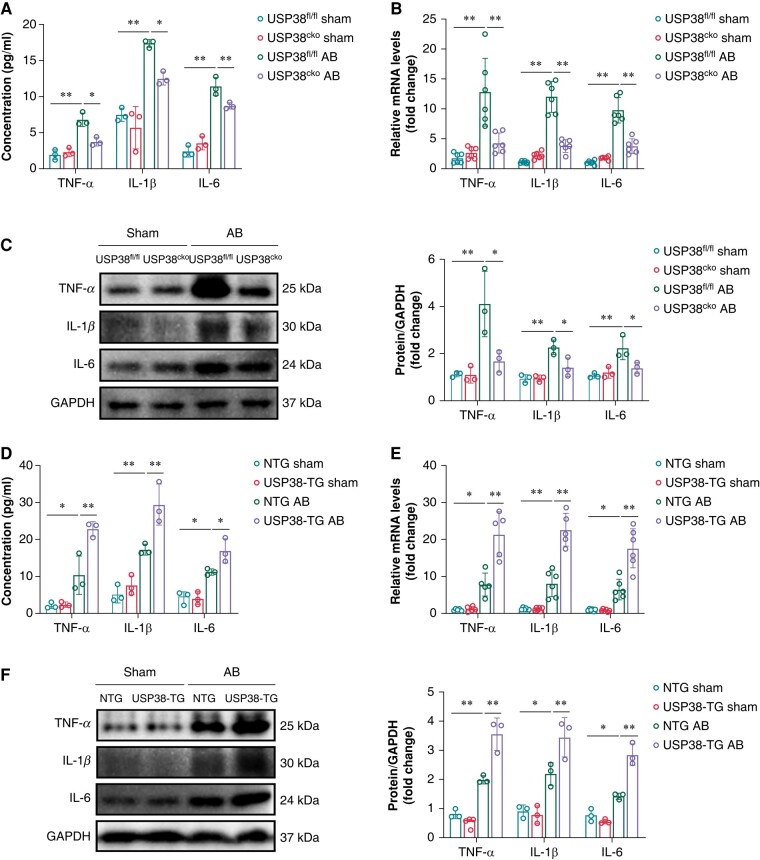
USP38 aggravates pressure overload-induced inflammatory responses. (*A*) The statistical analysis of the TNF-α, IL-1β, and IL-6 levels in serum of USP38^fl/fl^ and USP38^cko^ mice at 4 weeks after sham or AB surgery (*n* = 3). (*B*) The statistical analysis of mRNA expression of TNF-α, IL-1β, and IL-6 in left atrial tissues of USP38^fl/fl^ and USP38^cko^ mice at 4 weeks after sham or AB surgery (*n* = 5–6). (*C*) Representative western blot images and statistical analysis of the TNF-α, IL-1β, and IL-6 protein level in left atrial tissues of USP38^fl/fl^ and USP38^cko^ mice at 4 weeks after sham or AB surgery (*n* = 3). (*D*) The statistical analysis of the TNF-α, IL-1β, and IL-6 levels in serum of NTG and USP38-TG mice at 4 weeks after sham or AB surgery (*n* = 3). (*E*) The statistical analysis of mRNA expression of TNF-α, IL-1β, and IL-6 in left atrial tissues of NTG and USP38-TG mice at 4 weeks after sham or AB surgery (*n* = 5–6). (*F*) Representative western blot images and statistical analysis of the TNF-α, IL-1β, and IL-6 protein level in left atrial tissues of NTG and USP38-TG mice at 4 weeks after sham or AB surgery (*n* = 3). Data were calculated by one-way analysis of variance (Tukey’s multiple comparisons test). *P < 0.05; **P < 0.01.

To explore the role of USP38 in inflammation, we examined the level of inflammatory cytokines in serum and atrial tissues. Enzyme-linked immunosorbent assay results showed serum levels of TNF-α, IL-1β, and IL-6 were increased after pressure overload stimulation. However, USP38 deletion decreased those inflammatory cytokines expressions (*Figure 6A*). Consistently, quantitative reverse transcription polymerase chain reaction and western blot analysis showed that inflammatory cytokines expression after AB surgery was downregulated in USP38^cko^ mice compared with USP38^fl/fl^ mice (*Figure 6B* and *C*). Conversely, inflammation also was exacerbated after AB in USP38-TG group by increased levels of serum inflammatory cytokines and atrial tissue inflammatory marker proteins and transcription (*Figure 6D–F*).

**Figure 7:**
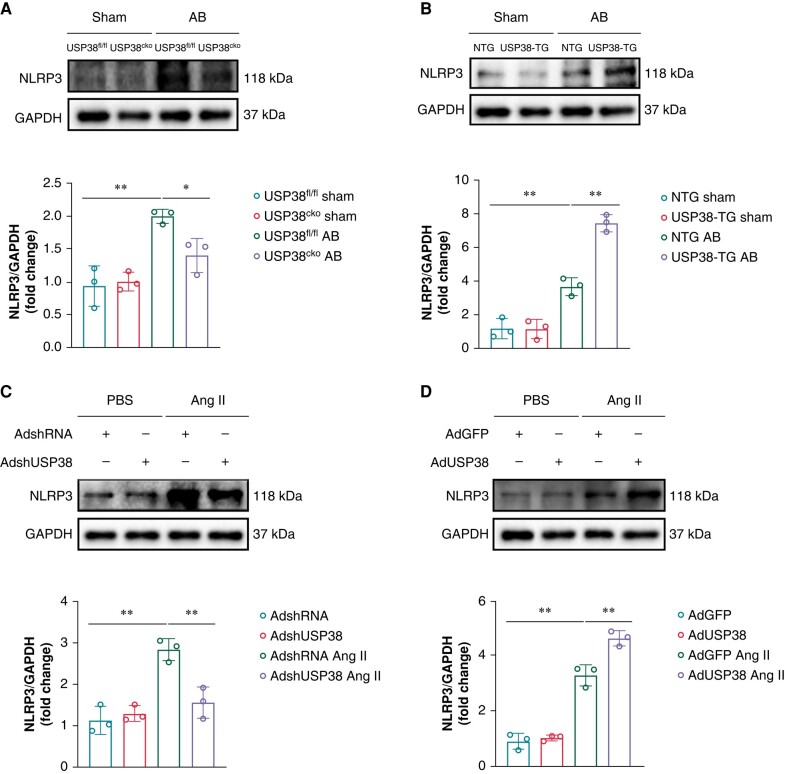
USP38 promotes the activation of NLRP3 under hypertrophic stimulation. (*A*) Representative western blot images and statistical analysis of NLRP3 in left atrial tissues of USP38^fl/fl^ and USP38^cko^ mice at 4 weeks after sham or AB surgery (*n* = 3). (*B*) Representative western blot images and statistical analysis of NLRP3 in left atrial tissues of NTG and USP38-TG mice at 4 weeks after sham or AB surgery (*n* = 3). (*C*) Representative western blot images and statistical analysis of NLRP3 in HL-1 cells that transfected with AdshRNA or AdshUSP38 for 24 h and treated with PBS or Ang II (1 μM) for 48 h (*n* = 3). (*D*) Representative western blot images and statistical analysis of NLRP3 in HL-1 cells that transfected with AdGFP (adenovirus-expressing green fluorescent protein) or AdUSP38 for 24 h and treated with PBS or Ang II (1 μM) for 48 h (*n* = 3). Data were calculated by one-way analysis of variance (Tukey’s multiple comparisons test). *P < 0.05; **P < 0.01.

Furthermore, our findings demonstrated that, after AB surgery, the elevated levels of NLRP3 were reduced in USP38^cko^ mice (*Figure 7A*), whereas USP38 overexpression led to further increase in the expression of NLRP3 (*Figure 7B*). Comparable results were also observed in the expression of NLRP3 in HL-1 cells (*Figure 7C* and *D*).

### Ubiquitin-specific protease 38 regulates nuclear factor-kappa B during atrial remodelling

**Figure 8:**
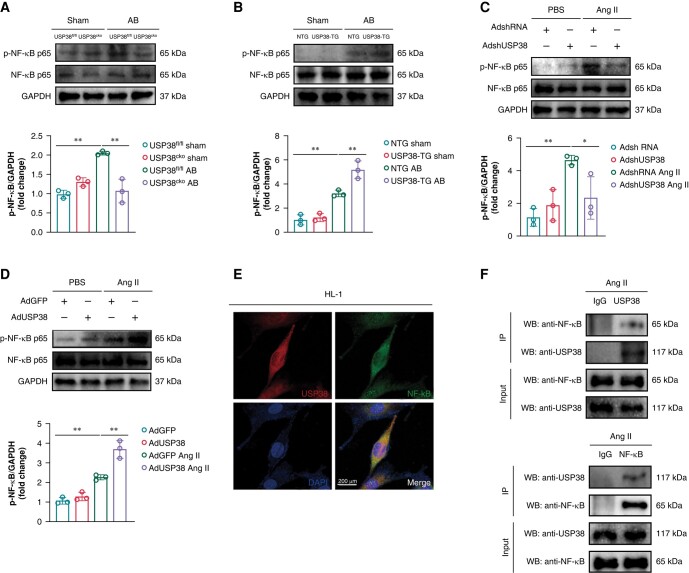
USP38 is involved in pressure overload-induced atrial remodelling by interacting with NF-κB. (*A*) Representative western blot images and statistical analysis of the p-NF-κB protein level in left atrial tissues of USP38^fl/fl^ and USP38^cko^ mice at 4 weeks after sham or AB surgery (*n* = 3). (*B*) Representative western blot images and statistical analysis of the p-NF-κB protein level in left atrial tissues of NTG and USP38-TG mice at 4 weeks after sham or AB surgery (*n* = 3). (*C*) Representative western blot images and statistical analysis of the p-NF-κB protein level in HL-1 cells that transfected with AdshRNA or AdshUSP38 for 24 h and treated with PBS or Ang II (1 μM) for 48 h (*n* = 3). (*D*) Representative western blot images and statistical analysis of the p-NF-κB protein level in HL-1 cells that transfected with AdGFP or AdUSP38 for 24 h and treated with PBS or Ang II (1 μM) for 48 h (*n* = 3). (*E*) Representative confocal images of the co-localization of USP38 and NF-κB in HL-1 cells treated with Ang II (1 μM) for 48 h (*n* = 3). (*F*) Endogenous immunoprecipitation analysis of the interaction between USP38 and NF-κB in HL-1 cells using anti-IgG, anti-USP38, or anti-NF-κB treated with Ang II (1 μM) for 48 h. Data were calculated by one-way analysis of variance (Tukey’s multiple comparisons test). *P < 0.05; **P < 0.01.

The NF-κB-mediated inflammation is a key mechanism of atrial structural and electrical remodelling. Therefore, we explored whether USP38 is involved in atrial remodelling by regulating NF-κB. Firstly, we found that USP38 deficiency obviously reduced the increased expression of p-NF-κB (Ser536) caused by pressure overload (*Figure 8A*), whereas USP38 overexpression further upregulated the level of p-NF-κB (*Figure 8B*). Similar results were found *in vitro* experiment. The transfected efficiency of AdshUSP38 and AdUSP38 was detected by western blots (see Supplementary material online, *Figure S4A* and *B*). In Ang II-treated cells, knockdown of USP38 decreased p-NF-κB expression (*Figure 8C*), whereas overexpression of USP38 further increased its expression (*Figure 8D*). Secondly, we also found the co-localization of USP38 and NF-κB in HL-1 cells under Ang II stimulation (*Figure 8E*). Finally, we detected the interaction of USP38 with NF-κB after Ang II administration by co-immunoprecipitation (*Figure 8F*). The results showed that USP38 antibody effectively precipitated NF-κB and vice versa.

### Ubiquitin-specific protease 38 reduces the ubiquitination level of nuclear factor-kappa B

**Figure 9:**
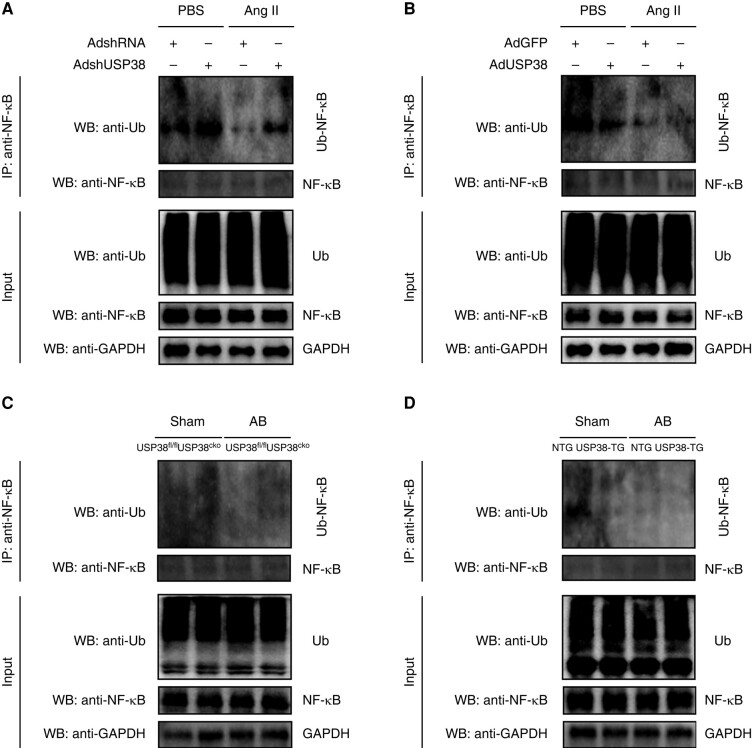
USP38 regulates the ubiquitination level of NF-κB. (*A*) Result of ubiquitination assays confirming the expression and ubiquitination of NF-κB in HL-1 cells that transfected with AdshRNA or AdshUSP38 and treated with PBS or Ang II followed by treatment with MG132 for 6 h before harvest. (*B*) Result of ubiquitination assays confirming the expression and ubiquitination of NF-κB in HL-1 cells that transfected with AdGFP or AdUSP38 and treated with PBS or Ang II followed by treatment with MG132 for 6 h before harvest. (*C*) Result of ubiquitination assays confirming the expression and ubiquitination of NF-κB in atrial tissue from USP38^fl/fl^ and USP38^cko^ mice after sham or AB surgery. (*D*) Result of ubiquitination assays confirming the expression and ubiquitination of NF-κB in atrial tissue from NTG and USP38-TG mice after sham or AB surgery.

In this section, we assessed the effect of USP38 on the ubiquitination of NF-κB. Firstly, we detected whether USP38 is associated with NF-κB ubiquitination in cellular level. We found that USP38 knockdown increased the ubiquitination level of NF-κB, whereas USP38 overexpression reduced its ubiquitination level (*Figure 9A* and *B*). Consistent with *in vitro* experimental results, USP38 also regulated NF-κB ubiquitination level in mouse atrial tissues (*Figure 9C* and *D*).

### Inhibition of nuclear factor-kappa B reduces ubiquitin-specific protease 38-mediated aggravation of aortic banding-induced atrial fibrillation

**Figure 10:**
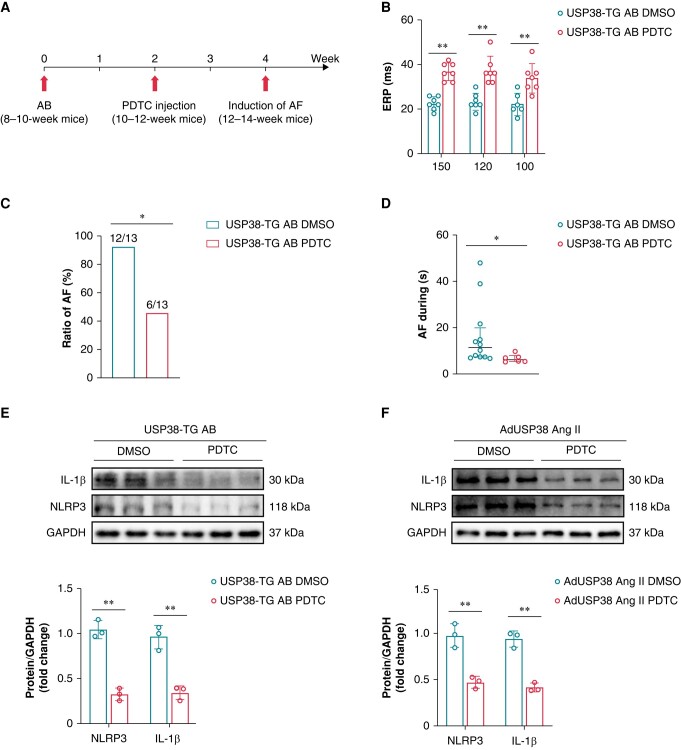
Inhibition of NF-κB reduces vulnerability to AF. (*A*) Protocol for injecting with PDTC in a mouse model of atrial remodelling. (*B*) ERP of the left atrium of USP38-TG DMSO and USP38-TG PDTC mice after AB surgery (*n* = 6–8). (*C*) Inducibility of AF of USP38-TG DMSO and USP38-TG PDTC after AB surgery (*n* = 13). (*D*) Duration of AF of USP38-TG DMSO and USP38-TG PDTC after AB surgery (*n* = 13). (*E*) Representative western blot images and statistical analysis of NLRP3 and IL-β in USP38-TG DMSO and USP38-TG PDTC mice after AB surgery (*n* = 3). (*F*) Representative western blot images and statistical analysis of NLRP3 and IL-β in HL-1 cells transfected with AdUSP38 and treated with Ang II with or without PDTC administration (*n* = 3). Data in *B, E and F* were calculated by Student’s *t*-test (unpaired, two-tailed, two groups). Data in *C* were calculated by Fisher’s exact test. Data in *D* were calculated by Mann–Whitney test. *P < 0.05; **P < 0.01.

To further explore whether USP38 is involved in AB-induced AF through NF-κB, we administered intra-peritoneal injection of PDTC (Sigma, USA) 2 weeks after AB surgery in USP38-TG mice (*Figure 10A*), which has been shown to inhibit NF-κB activation. The results showed an increased ERP and a reduced susceptibility and duration of AF following PDTC administration (*Figure 10B–D*). Meanwhile, we found that the expression of NLRP3 and IL-1β was downregulated after PDTC treatment *in vivo* and *in vitro* experiments (*Figure 10E* and *F*).

## Discussion

The major findings from our current study uncover that USP38 acts as a positive regulator in pressure overload-induced AF. Ubiquitin-specific protease 38 overexpression markedly exacerbates atrial structural and electrical remodelling, increasing vulnerability to AF. Conversely, we used cardiac-specific USP38 knockout mice and found that atrial remodelling and susceptibility to AF are significantly improved in USP38 knockout mice. Mechanistically, USP38 interacts with NF-κB and mitigates the ubiquitination level of NF-κB, which promotes the activation of NLRP3. These findings strongly suggest that USP38 accelerates the progression of adverse atrial remodelling induced by pressure overload by activating the NF-κB/NLRP3 signalling.

Previous study has shown that vulnerability to AF increases in pressure overload animal model.^12^ The increased vulnerability to AF is correlated with a shorter atrial ERP (AERP).^30^ In our study, the shortening AERP and the increasing susceptibility to AF were found in pressure overload-induced mouse model.

Due to the many hazards of AF to human health, more attention should be paid to the pathogenesis of AF. The current treatment options for AF are limited, and the side-effects are obvious. Previous studies have demonstrated that atrial structural and electrical remodelling plays a fundamental role in the trigger and perpetuation of AF.^31^ Atrial structural remodelling is usually manifested atrial enlargement and atrial fibrosis, causing delayed conduction and local heterogeneous conduction, which creates the substrate for AF.^31–33^ It is worth noting that chronic pressure overload promotes collagen synthesis, causing excessive deposition of extra-cellular matrix proteins in the atrial tissue.^34^ Moreover, the atrial diameter increases under pressure overload stimulation,^34^ and the atrial enlargement in patients is associated with the occurrence of AF.^33^ In this study, we noticed that pressure overload caused the enlargement of LAD was more obvious in USP38-TG mice compared with USP38-NTG mice. Moreover, the degree of atrial fibrosis was more severe in USP38-TG mice under pressure overload stimulation, suggesting that USP38 overexpression further exacerbates pressure overload-induced atrial structural remodelling.

In addition, atrial electrical remodelling is mainly caused by abnormal expression and function of various ion channels,^27,35^ among which dysregulated calcium homoeostasis is a greater risk factor for AF.^36^ The maintenance of calcium homoeostasis is closely related to calcium-handling proteins, which are essential for the contraction and relaxation of cardiomyocyte.^35^ For example, RyR2 regulates intra-cellular Ca^2+^ release from SR, and subsequently SR/endoplasmic reticulum Ca^2+^-ATPase (SERCA2a) is responsible for sequestering Ca^2+^ into SR.^37^ Phospholamban decreases Ca^2+^ reuptake into SR by inhibiting SERCA2a.^37^ The abnormal expression and function of calcium-handling proteins are prone to cause systolic and diastolic dysfunction and arrhythmias.^38^ Our previous studies have shown that pressure overload causes elevated levels of RyR2 and PLB phosphorylation and decreased level of SERCA2a, all of which contributes to the reduction of SR Ca^2+^ content and intra-cellular Ca^2+^ overload, increasing arrhythmogenic potential.^27^ Consistently, our study showed an increased expression of p-RyR2 at Ser2808 and p-PLB at Thr17 and decreased expression of SERCA2a after AB surgery, which was further worsened in USP38 overexpression heart. Thus, USP38 deletion improved atrial electrical remodelling induced by pressure overload through regulating calcium homoeostasis.

Currently, accumulating studies have demonstrated that abnormal Ca^2+^ handling is a key mechanism of inflammation-related AF.^4,39^ Meanwhile, inflammatory cytokines serve as crucial biomarkers that can predict the incidence and prognosis of AF.^39^ Pressure overload leads to an increase in a series of inflammatory cytokines in the heart, such as TNF-α, IL-1β, and IL-6,^40,41^ which not only accelerate the progression of pathological cardiac remodelling but also promote the secretion of inflammatory cytokines by recruiting more inflammatory cells to the heart.^41^ Overexpression of TNF-α causes abnormal Ca^2+^ release and handling in the atrial myocardium and then increases vulnerability to AF.^42,43^ Pressure overload did not lead to an increase in the induction rate of AF in IL-1β knockout mice, suggesting that IL-1β plays a vital role in pressure overload-induced AF.^44^ Interleukin-6 has been found to be involved in atrial electrical remodelling by regulating cardiac connexin.^45^ Recently, Chin *et al*. identified a newly inflammatory cytokine macrophage migration inhibitory factor that is highly expressed in patients with AF. It causes Na^+^ and Ca^2+^ dysregulation in pulmonary vein cardiomyocytes and promotes the genesis of AF during inflammation.^46^ Furthermore, NLRP3 inflammasome activation in atrium cardiomyocytes enhances some ion channel subunits expression and increases vulnerability to AF, accompanied by the increase of IL-1β and IL-18.^13^ Acute application of IL-1β promotes SR Ca^2+^ release in HL-1 cells through activating NLRP3 signalling.^47^ Notably, NF-κB has been reported as a crucial transcription factor that promotes the transcription of many inflammatory cytokines during atrial remodelling.^12,48^ Activation of NF-κB/NLRP3 signalling pathway increases the level of inflammatory cytokines and accelerates the progression of AF.^11,49^ Ubiquitination plays a critical role in NF-κB inactivation and degradation.^50,51^ Our data showed that USP38 binds to and deubiquitinates NF-κB and a marked activation of NF-κB in USP38 overexpression heart after AB surgery, accompanied by the increased expression of TNF-α, IL-1β, IL-6, and NLRP3. In addition, inhibition of NF-κB decreased the expression of NLRP3 and IL-1β and reduced susceptibility to AF in USP38-TG mice after AB surgery. This indicates that USP38 promotes NF-κB/NLRP3 signalling-mediated inflammatory cytokines expression, exacerbating pressure overload-induced atrial remodelling.

## Limitation

The current study has some limitations. Firstly, sex differences are associated with cardiac function and morphology in pressure overload-induced cardiac remodelling model. In this study, we only used male mice for experiments, but the regulation of USP38 for female mice has not been studied. Secondly, while cardiac function was evaluated by echocardiography, haemodynamic analyses might provide more beneficial insights into cardiac function in pressure overload models. Thirdly, the left heart and right heart were involved sequentially after AB surgery. Although our study mainly explored the changes in the left atrium, the impact on the right atrium was not assessed. Finally, a large sample size does provide a better description of the experimental phenomenon. The experimental sample of some data in this study only keeps to the minimum necessary to answer scientific questions. Therefore, future studies of this disease model should address sex differences and right atrial effects in this disease model, as well as the relationship between of experimental animal sample size and animal welfare.

## Conclusion

In conclusion, USP38 promotes inflammatory AF induced by pressure overload. This is due to USP38-exacerbating pressure overload-induced atrial structural and electrical remodelling through NF-κB/NLRP3-mediated inflammation. Therefore, USP38 may serve as a new therapeutic target for pressure overload-induced AF.

## Supplementary Material

euad366_Supplementary_Data

## Data Availability

The data underlying this article will be shared on reasonable request to the corresponding author.
