# Supplementary material for: Ubiquitin-specific protease 38 promotes inflammatory atrial fibrillation induced by pressure overload
Source: Europace. 2024 Jan 29;26(1):euad366. doi: 10.1093/europace/euad366 (PMC10823351; doi:10.1093/europace/euad366)
Supplement: euad366_Supplementary_Data [file euad366_supplementary_data.docx]

1. **Supplementary Tables**

**Table S1 Sequence of primers used for quantitative real-time polymerase chain reaction (qRT-PCR).**

| Genes | Species | Sequence (5’ → 3’) |
| --- | --- | --- |
| TNF-α | Mouse | (Forward)-GGTGCCTATGTCTCAGCCTCTT  (Reverse)-GCCATAGAACTGATGAGAGGGAG |
| IL-1β | Mouse | (Forward)-TGGACCTTCCAGGATGAGGACA  (Reverse)- GTTCATCTCGGAGCCTGTAGTG |
| IL-6 | Mouse | (Forward)-CAAAGCCAGAGTCCTTCAGAG  (Reverse)-GCCACTCCTTCTGTGACTCC |
| Collengen I | Mouse | (Forward)-AGGCTTCAGTGGTTTGGATG  (Reverse)-CACCAACAGCACCATCGTTA |
| Collengen III | Mouse | (Forward)-CCCAACCCAGAGATCCCATT  (Reverse)-GAAGCACAGGAGCAGGTGTAGA |
| TGF-β | Mouse | (Forward)-GTGGCTGAACCAAGGAGACG  (Reverse)-AGGTGTTGAGCCCTTTCCAG |
| GAPDH | Mouse | (Forward)-ACTCCACTCACGGCAAATTC  (Reverse)-TCTCCATGGTGGTGAAGACA |

**Table S2 Primary antibodies for Western-blots**

| Primary antibodies | Source organism | Producer | Number |
| --- | --- | --- | --- |
| USP38 | Rabbit | Proteintech | 17767-1-AP |
| Collagen I | Rabbit | Affinity | AF7001 |
| Collagen III | Rabbit | Affinity | AF5457 |
| TGF-β | Rabbit | Affinity | AF1027 |
| RyR2 | Rabbit | Affinity | AF0015 |
| p-RyR2 | Rabbit | Affinity | AF7475 |
| SERCA2a | Rabbit | Affinity | DF6240 |
| PLB | Rabbit | Affinity | AF7778 |
| p-PLB | Rabbit | CST | #8496 |
| TNF-α | Rabbit | CST | #11948 |
| IL-1β | Rabbit | Abcam | ab9722 |
| IL-6 | Rabbit | CST | #12912 |
| NF-κB | Rabbit | CST | #8242 |
| p- NF-κB | Rabbit | CST | #3031 |
| IgG | Rabbit | CST | 8726 |
| NF-κB | Mouse | Abcam | #6956 |
| NLRP3 | Rabbit | CST | #15101 |
| Ubiquitin | Rabbit | CST | #3933 |
| GAPDH | Rabbit | CST | #5174 |

1. **Supplementary Figures**

**Figure S1**


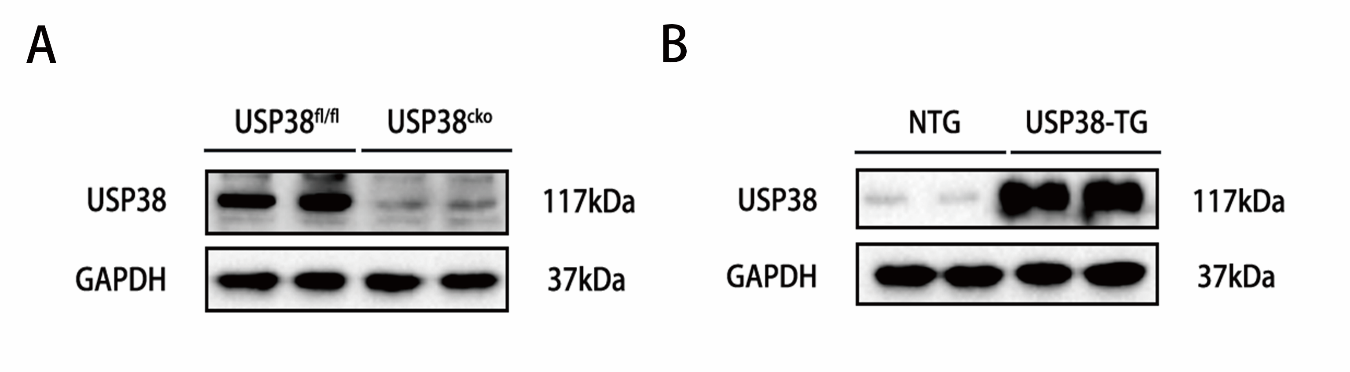


**Figure S1. The efficiency of cardiac-specific USP38 knockout and transgene in mice.** **(A)** Representative immunoblotting of USP38 protein in the atrial tissues from USP38^fl/fl^ and USP38^cko^ mice (n=4). **(B)** Representative immunoblotting of USP38 protein in the atrial tissues from NTG and USP38-TG mice (n=4).

**Figure S2**


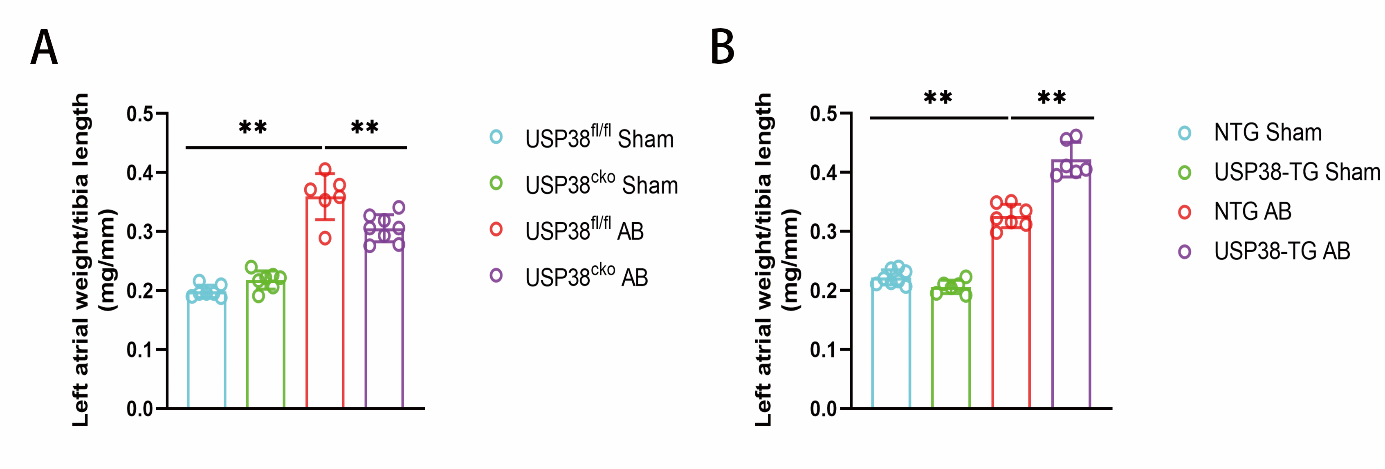


**Figure S2. USP38 alteres mouse left atrial weight after AB surgery. (A)** The statistical analysis of left atrial weight/tibia length of USP38^fl/fl^ and USP38^cko^ mice at 4 weeks after sham or AB surgery (n=6-8). **(B)** The statistical analysis of left atrial weight/tibia length of NTG and Usp38-TG mice at 4 weeks after sham or AB surgery (n=6-8). The *p*-value was calculated by one-way analysis of variance (Tukey’s multiple comparisons test). **P* < 0.05, ***P* < 0.01.

**Figure S3**


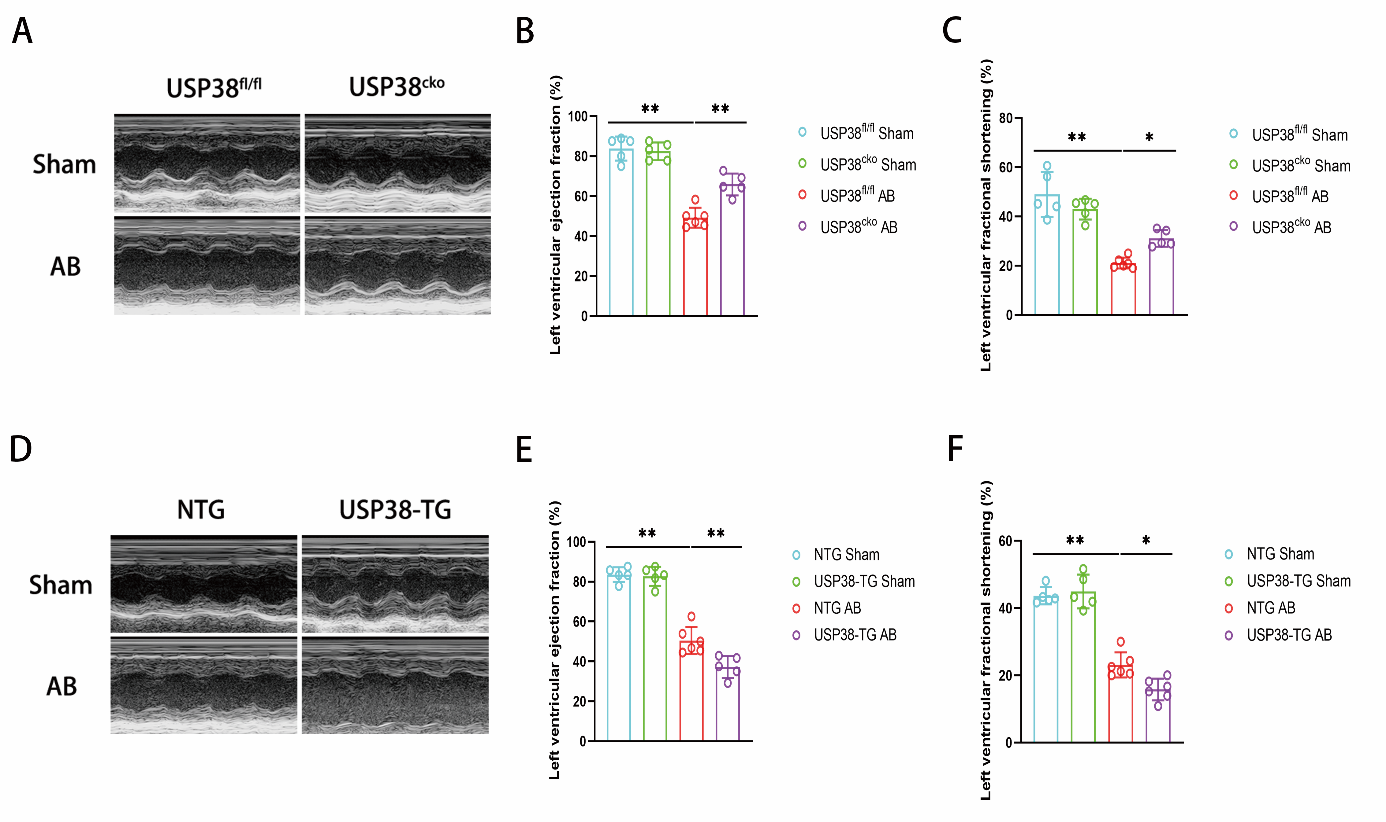


**Figure S3. USP38 aggravates pressure overload induced ventricular function.** **(A)** Representative echocardiographic images and statistical analysis of **(B)** left ventricular ejection fraction and **(C)** left ventricular fraction shortening of USP38^fl/fl^ and USP38^cko^ mice at 4 weeks after sham or AB surgery (n=5-6). **(D)** Representative echocardiographic images and statistical analysis of **(E)** left ventricular ejection fraction and **(F)** left ventricular fraction shortening of NTG and USP38-TG mice at 4 weeks after sham or AB surgery (n=5-6). The *p*-value was calculated by one-way analysis of variance (Tukey’s multiple comparisons test). **P* < 0.05, ***P* < 0.01.

**Figure S4**


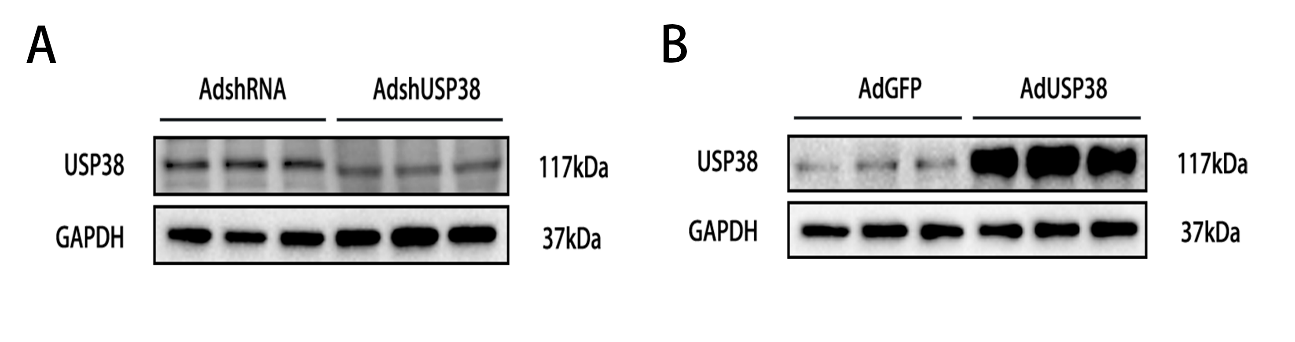


**Figure S4. The transfection efficiency of** **AdshUSP38 and AdUSP38 in HL-1 cells.** **(A)** Representative immunoblotting of USP38 protein in cells transfected with AdshRNA or AdshUSP38 (n=3). **(B)** Representative immunoblotting of USP38 protein in cells transfected with AdGFP or AdUSP38 (n=3).
